# Exergaming Can Be a Health-Related Aerobic Physical Activity

**DOI:** 10.1155/2019/1890527

**Published:** 2019-06-04

**Authors:** Jacek Polechoński, Małgorzata Dębska, Paweł G. Dębski

**Affiliations:** ^1^Department of Tourism and Health-Oriented Physical Activity, The Jerzy Kukuczka Academy of Physical Education, Katowice, 40-065, Poland; ^2^Chair and Clinical Department of Psychiatry, School of Medicine with the Division of Dentistry in Zabrze, Medical University of Silesia in Katowice, Poland

## Abstract

The purpose of the study was to assess the intensity of aerobic physical activity during exergame training sessions with a moderate (MLD) and high (HLD) level of difficulty of the interactive program “Your Shape Fitness Evolved 2012” for Xbox 360 Kinect in the context of health benefits. The study involved 30 healthy and physically fit students. During the game, the HR of the participants was monitored using the Polar M400 heart rate monitor. The average percentage of maximum heart rate (%HRmax) and heart rate reserve (%HRR) during the game was calculated and referred to the criterion of intensity of aerobic physical activity of American College of Sports Medicine and World Health Organization health recommendations. During the MLD training, the participants achieved on average 69.6 ± 8.7%  HR_max_ and 57.0 ± 11.9% HRR (moderate intensity), while performing HLD exercises, they achieved 78.9 ± 8.1%  HR_max_ and 70.2 ± 11.3% HRR (vigorous intensity). The time spent in recommended moderate-to-vigorous intensity during 15-min exergame session was 14.6 min (97,1%) for MLD and 14.8 min (99%) for HLD. The intensity of aerobic PA during exergame “Your Shape Fitness Evolved 2012” both medium and high level of difficulty almost all the training sessions was at the level recommended for health benefits. Active video games, especially exergames, containing an element of physical activity, can be used to increase the weekly dose of PA in the direction recommended for health benefits.

## 1. Introduction

The development of modern technology is considered one of the reasons for declining levels of physical activity in everyday life of people around the world in recent decades [1]. The lack of movement (hypokinesia) is the main cause of the incidence of noncommunicable chronic diseases. According to the recent statistics these diseases cause 71% of all deaths per year [2]. Therefore, activities for health promotion are currently focused on searching for tools popularizing physical activity among contemporary people, tailored to their interests, physical abilities, and leisure time budgets [3].

Currently, video games are one of the most common and dynamically developing forms of free-time activity regardless of age. This form of recreation is declared by 58% of the North American population [4, 5] and 53% of the European population [6]. As a result, in the past few years, a great development in the video game market has been observed. Besides the traditional video games, more and more active video games (AVGs), in which the user controls the game by moving their whole body, are rising. Such kind of games results in higher motor activation in players than during typical games [7]. Among the AVGs, there are further separated exergames, various training programs, often with the assistance of a virtual trainer, whose goal is physical activity (PA) [8].

In connection with the above, the research interest in verifying the level of physical activity during differentiated AVGs [9–12] increased, as well as the possibility of using them to promote health behaviors, including regular physical activity [13–18]. The results of monitoring the parameters of physical exercise during many AVGs have shown that the values of some of them are at the level recommended for health by international organisations [19, 20]. At the same time, it was noticed that the intensity and, consequently, the caloric cost of such kind of physical activity are very diverse, depending on the form of movement, level of involvement of the muscle apparatus (limb vs. the whole body), level of difficulty of the game, and experience in playing [11, 21–23]. Therefore, it seems important to observe the physiological response to physical efforts undertaken during popular AVGs in order to select those with a pro-health character.

The results of many studies indicate a positive effect of physical efforts occurring during AVGs on the improvement of health [24–26]. It was observed that, thanks to the high attractiveness of games of this type, players are able to perform longer physical activity in an interactive form, compared to classic exercises, which may translate into better health effects [25]. Research is also carried out on the effectiveness of AVGs in secondary prevention [27–29], including in patients after stroke [30], suffering from depression [31], multiple sclerosis [32, 33], and cancers [34].

It is well known that the time spent on playing different computer games in the population of different ages increases. Physical effort during AVGs can raise health-enhancing PA, so this form of recreation could be a supplement to the daily dose of recommended physical activity. In addition, the high level of pleasure experienced during AVGs increases the attractiveness of physical activity undertaken in its course and consequently may more effectively motivate to take it systematically [35, 36].

Today, there are many consoles that allow practising exergames. The most popular ones include Nintendo Wii, Play Station (Move), and Xbox (Kinect). The last of the mentioned devices is particularly noteworthy. The Xbox Kinect gaming console (Microsoft Corp) consists of the Xbox video game console and a self-adjustable camera, which acts as a sensor to detect whole body movements. This gaming system provides a controller-free type of gaming in which the individual controls the games using his or her body movements. Overall, exergames focusing on the lower body and the whole body expend more energy than those focusing on the upper body alone [17].

The aim of the study was to assess the level of intensity of aerobic PA of selected training sessions with a moderate (MLD) and high (HLD) level of difficulty of the interactive program “Your Shape Fitness Evolved 2012” for Xbox 360 Kinect for the possibility of achieving health benefits. The results obtained in the course of the research were referred to the World Health Organization and the American College of Sports Medicine health recommendations. Both the average intensity levels of PA during MLD and HLD were assessed, as well as the time spent in moderate-to-vigorous PA (MVPA) during 15-minute bouts of exergaming with the Xbox Kinect game console.

## 2. Materials and Methods

### 2.1. Participants

The study involved 30 healthy and physically fit male students of the Academy of Physical Education in Katowice. The characteristics of the group are shown in Table 1. Participants were excluded if they were taking any medications affecting heart rate or had any physical limitations affecting exercise (pregnancy, injury, etc.). They had no history of seizures or epilepsy, and they were informed of the product safety information. All subjects were familiarized with the aim of monitoring of physical activity and conducting measurements and forms of use of their results. They did not have any previous experience with the exergame used in this study.

### 2.2. Procedures

#### 2.2.1. Your Shape Fitness Evolved 2012

Among the many popular exergames for Xbox Kinect, such as Zumba Fitness, EA Sports Active, or UFC Personal Trainer, Ubisoft's Your Shape Fitness Evolved 2012 is positively distinguished, which not only accurately transfers the movements of exercising people to the screen, but also offers quite a substantial and varied set of exercises and activities. The Player Projection system helps in this. It is a tool that can very accurately reproduce the shape of the player's body and display it on the screen. Then, it follows the player's movements during the exercise and keeps them informed about any mistakes.

#### 2.2.2. Experimental Trial

**Figure 1:**
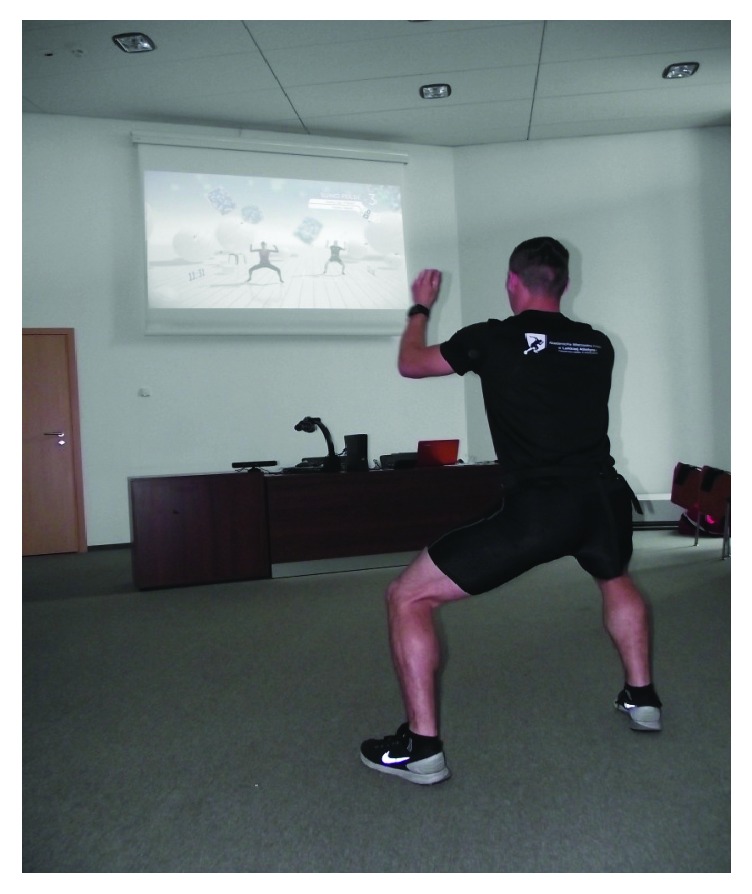
Test place: the distance between the subject and the Xbox Kinect controller, 2,5 m. Source: our own elaboration.

The students participated in two 15-minute exergaming sessions included in the “Workouts Cardio” section of Your Shape Fitness Evolved 2012 for Xbox 360 Kinect. The task of the respondents was to imitate the movements of the virtual trainer (Figure 1).

The study was conducted on two levels of difficulty: medium (MLD, “Break a Sweat E” training) and high (HLD, “Break a Sweat G” training). Between the training sessions, there was a 20-minute rest break. Previously, the students were instructed in the use of the application. The Break a Sweat version E consisted of 6 sets of 3 exercises each; version G consisted of 2 sets of 9 exercises (Table 2).

The research was carried out before noon in a quiet laboratory room equipped with a multimedia projector, a 118-inch screen, and Xbox 360 Kinect console from Microsoft. The test subjects were located approximately 4.5 m from the screen. They had a space allowing for the free movement of the whole body and limbs (about 25 m^2^). The subjects exercised in loose sports outfits. The tests were performed individually, without the presence of people other than the researcher (minimisation of factors that could affect the heart rate, HR). The students proceeded to gaming without a prior warm-up to avoid any possible impact on the measurement results.

During the game, the HR of the study participants was monitored using the Polar M400 heart rate monitor cooperating with the transmitter (H7) placed with a flexible chest strap. The intensity of physical exercise during the game was determined on the basis of the average percentage of maximum heart rate (%HRmax) and heart rate reserve (%HRR) of the participants. The level of intensity was estimated according to the classification of PA-intensity proposed by the American Heart Association, according to which an intensity of ≥ 50% HRmax or ≥45% HRR was considered moderate and ≥70% HRmax or ≥60% HRR was vigorous [37]. The HRmax values were calculated from the Tanaka formula [38] while the HRR values were calculated by using the Karvonen formula [39]. The data obtained in this way was referred to the criterion of intensity of aerobic physical activity recommended by the American College of Sports Medicine and the World Health Organization. It was assumed that moderate and vigorous intensity efforts are beneficial to health [19, 20].

The exercise intensity was also categorised into HR zones, using the Polar Flow training analysis tool. Both the absolute (in minutes) and the relative (in percent) times of HR spent in each of the six zones were estimated: <50% HRmax, 50-59% HRmax, 60-69% HRmax, 70-79% HRmax, 80-89% HRmax, and ≥90% HRmax. The calculations were made for each of the selected training programs (MLD, HLD), which allowed comparing them in the context of the intensity.

### 2.3. Ethics

The study procedures were reviewed and approved by institutional review board. It was conducted in accordance with the Declaration of Helsinki, and the procedures were approved by the Research Ethics Committee of the Jerzy Kukuczka Academy of Physical Education in Katowice. All participants took part in the study voluntarily and could discontinue their participation at any time. They have provided written consent for the use of information collected during examination.

### 2.4. Statistical Analysis

For the statistical analysis, the Statistica v. 13 software (TIBCO Software Inc.) was used. Arithmetic means, standard deviations, and the differences between the mean values were calculated. The normality of data distribution was assessed with the Shapiro-Wilk test. The statistical significance of the differences between the results was determined by Student's t-test.

## 3. Results

**Figure 2:**
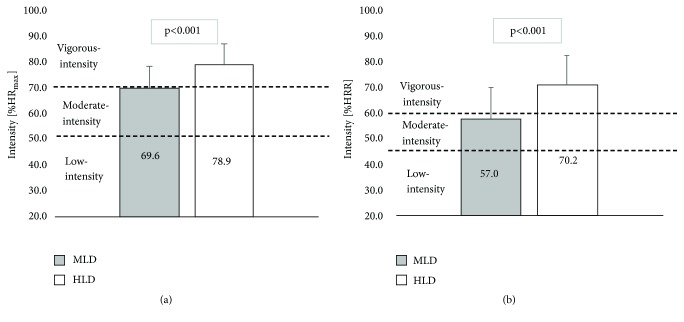
Physical activity intensity during exergaming shown in %HR_max_. (a) %HRR (b). MLD: moderate level of difficulty; HLD: high level of difficulty; HR_max_: maximum heart rate. Error bars represent SD (n=30).

The mean exercise heart rate during the HLD program (150.9 ± 15.5 bpm) was 17.8 bpm higher compared to MLD (133.1 ± 16.6 bpm). The demonstrated difference turned out to be statistically significant (p <0.001). The average PA intensity expressed by %  HR_max_ and % HRR during both training programs was at the level recommended for health in all participants. In the case of MLD training, the participants achieved on average 69.6 ± 8.7%  HR_max_ and 57.0 ± 11.9% HRR (moderate intensity), while, performing HLD exercises, they achieved 78.9 ± 8.1%  HR_max_ and 70.2 ± 11.3% HRR (vigorous intensity). Differences in results recorded during both trainings amounted to 9.3%  HR_max_ and 13.2% HRR and were statistically significant (p <0.001) (Figure 2).

**Figure 3:**
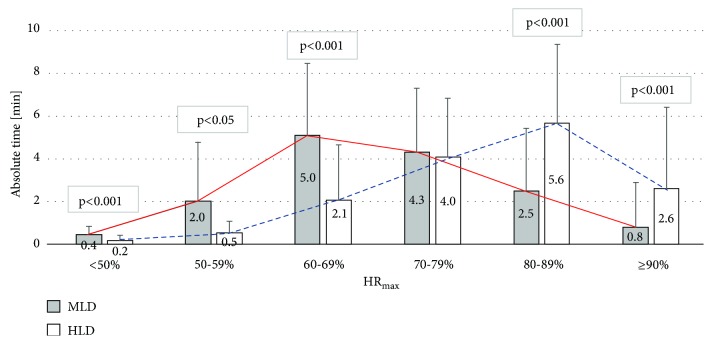
Absolute time spent in different heart rate (HR) zones during exergaming. MLD: moderate level of difficulty; HLD: high level of difficulty; HR_max_: maximum heart rate. Error bars represent SD (n=30).

Assessing the health-enhancing nature of PA, it is worth determining not only the average level of its intensity, but also the time for which the person doing the exercises stays in the range of training loads recommended for health. The participants spent in MVPA (above 50%  HR_max_) 14.6 min (97,1%) during MLD and 14.8 min (99%) during HLD out of 15-min training session. Analyzing the time spent in each of the heart rate zones, the biggest difference between the intensity during MLD and HLD was found in areas: 60-69% HR max and above 80%  HR_max_ (Figure 3).

## 4. Discussion

The aim of the work was to verify the PA intensity of the aerobic type of selected training units of the interactive program “Your Shape Fitness Evolved 2012” for Xbox 360 Kinect in relation to the recommended values for health benefits by WHO [20] and ACSM [19]. The results of our own research showed that the intensity of aerobic PA during both MLD and HLD exercise programs expressed by %HR_max_ and %HRR was usually moderate or vigorous. The participants spent in MVPA during a 15-minute MLD training session 14.6 min (97.1%) and during HLD one - 14.8 min (99%). It can, therefore, be said that exergaming “Your Shape Fitness Evolved 2012” on both tested levels of difficulty (MLD - “Break a Sweat E” and HLD training “Break a Sweat G”) guarantees PA of an intensity considered as prohealth.

However, not all AVGs that work with the Xbox Kinect console are intense enough to let the player perform physical effort that is recommended for health. The results of research aimed at verifying PA parameters during River Rush showed that the intensity of the effort was too low for health benefits to occur [9]. In this case, it was probably conditioned by the rather static plot of the game, which stimulated users mainly to body balancing and sporadic jumps. The low PA intensity could also result from the short duration of the gaming experience (5 minutes), and/or the selected game, which was focused on adventure.

Also, studies verifying the intensity of various exergames on another popular console enabling participation in AVGs, the Nintendo Wii, show varied results in terms of their pro-health character. Some exergames require less physical effort than that recommended by the WHO for health benefits (<3 METs) [7, 40], while others may be considered to have a health-enhancing intensity level [41–44]. For example, the average intensity of aerobic training (expressed in% HRR) demonstrated in the study of students engaged in Wii Fit aerobics activity by Mullins et al. [22] was almost two times lower (30% HRR) than during Your Shape Fitness Evolved 2012 AVG on moderate difficulty (57.0 ± 11.9% HRR) and two and a half times lower than estimated during the game on a high level of difficulty (70.2 ± 11.3% HRR), thus allowing health benefits to be obtained only by people with low levels of physical fitness, leading a typically sedentary lifestyle [19]. In the case of our study results, however, it was in the range of intensity commonly recommended for health benefits.

It should be remembered that, in addition to the criterion of health-oriented aerobic PA intensity, WHO and ACSM defined a minimum single bout of that type of effort (uninterrupted 10 minutes) and its weekly volume (150 min MPA or 75 min VPA or equivalent) [20], whereas ACSM recommends a specific frequency (5 times x 30 min MPA or 3 times x 25 min VPA or equivalent) [19], on the basis of which we can determine the pro-health character of this type of activity. In view of the above, wishing to fill in the above recommendation criteria, a one-time exergame effort should last at least 10 minutes, which is possible, among others, thanks to “Workouts Cardio” exercise units of Your Shape Fitness Evolved 2012 lasting on average for 15 minutes. The option of lengthening the training session also allows achieving the ACSM's recommended volume of PA (30 min MPA, 25 min VPA or equivalent). In the case of trainings tested in own research, for example, a 25-minute game on HLD three times a week allows meeting the above criteria.

International organizations related to public health recommend reducing the use of on-screen media, which in the general opinion is associated with passive spending of free time. However, active video games, especially exergames, containing an element of physical activity, can be used to increase the weekly dose of PA in the direction recommended for health benefits. Exergaming can be a particularly important tool for increasing health-oriented PA among children and adolescents, among whom the percentage of those with adequate PA level is low [7, 45, 46].

Among the most frequently declared reasons for the lack of regular participation in physical activity by various social groups, the most important are the following: not having enough time to exercise, finding it inconvenient to exercise, lack of self-motivation and finding exercises boring [47]. Therefore, it is necessary to look for forms of PA that will be attractive to the practitioners and will influence their motivation to act. There is scientific evidence that those who practice exergames find it satisfying [7, 17, 41]. Part of the research indicates an even greater level of enjoyment and increase in positive emotions during different forms of PA during AVGs compared to traditional ones [10, 23, 48]. Graves et al. 2014 [7] showed a high level of enjoyment during exergame despite a relatively high intensity of physical exercise during the game. The research conducted by Oh et al. 2016 [49] shows that exergaming activities are psychologically enjoyable pursuits for college-aged individuals that can help increase their physical health and quality of life. Therefore, it seems to be an effective tool to overcome the above-mentioned barriers to PA, contributing to the popularization of this healthy behavior and, consequently, to improving public health.

The limitation of our research is the small study group. The research was conducted in the group of healthy and physically fit students and tested a selected AVG. We are aware that its results do not give a complete answer to the question asked in the manuscript title. In order to get a full answer further comprehensive research in this area is needed. The findings of this manuscript should be investigated in different population groups (e.g., children, elderly, and disabled) and various active video games.

## 5. Conclusions

The study results indicate that the intensity of aerobic PA during Xbox Kinect exergame “Your Shape Fitness Evolved 2012” with both a medium and high level of difficulty was at the level recommended by WHO and ACSM for health benefits (moderate-to-vigorous). During MLD play, participants achieved on average 69.6 ± 8.7%  HR_max_ and 57.0 ± 11.9% HRR (moderate intensity), while. during HLD, they achieved 78.9 ± 8.1%  HR_max_ and 70.2 ± 11.3% HRR (vigorous intensity). Almost all the 15-minute exergaming session, the intensity of the effort remained moderate-to-vigorous, 14.6 min (97%) for MLD and 14.2 min (98.9%) for HLD.

The findings suggest that active video games, especially exergames, containing an element of physical activity, can be used to increase the weekly dose of PA in the direction recommended for health benefits. However, the availability of various types of exergames and consoles on the market leads to further research aimed at identifying those devices and software that will enable users to participate in PA compliant with health recommendations.

## Figures and Tables

**Table 1:** Participants' characteristics.

Characteristics	Total (n=30)
Age [years]	23.8 ± 1.3
Weight [kg]	76.2 ± 9.7
Height [cm]	177.9 ± 7.3
Body mass index [kg/m^2^]	24.1 ± 2.4
Heart Rate Rest (HR_rest_)*∗*[bpm]	55.8 ± 6.5
Maximum Heart Rate (HR_max_) [bpm]	191.4 ± 0.9

*∗*estimated using the formula 208 - 0.7 x age [38].

**Table 2:** Description of Break a Sweat exercises and repetitions.

Version E		Version G	
Exercises	Repetitions	Exercises	Repetitions
Power Jog	16	Flying Jog	16
Squat Punch	16	Oblique Swing	24
Slide Knee Cross	16	Oblique Swing	24
Sumo Pulse	8	Jab Knee-up	8
Knee up Cross	16	Triple Run Punch	16
Sumo Pulse	8	Slide Jump	8
X-Jog	16	Punch Side-Leap	16
Shuffle Cross Punch	8	Plyo Leg Curl	8
Jumping Jack Punch	8	Shuffle Cross Punch	8
Power Jog	16		
Squat Punch	16		
Slide Knee Cross	16		
Sumo Pulse	8		
Knee up Cross	16		
Sumo Pulse	8		
X-Jog	16		
Shuffle Cross Punch	8		
Jumping Jack Punch	8		

## Data Availability

The data used to support the findings of this study are available from the corresponding author upon request.
